# Exercise as a Molecular Therapeutic Strategy in Metabolic Syndrome: Integrating Cellular Signaling, Organ Crosstalk, and Clinical Translation—A Narrative Review

**DOI:** 10.3390/cimb48080850

**Published:** 2026-08-21

**Authors:** Héctor Fuentes-Barría, Raúl Aguilera-Eguía, Miguel Alarcón-Rivera, Cherie Flores-Fernández

**Affiliations:** 1 Centro de Investigación en Medicina de Altura (CEIMA), Universidad Arturo Prat, Iquique 1110939, Chile; 2 Departamento de Salud Pública, Facultad de Medicina, Universidad Católica de la Santísima Concepción, Concepción 3349001, Chile; raguilerae@ucsc.cl; 3 Dirección de Investigación, Universidad Bernardo O’Higgins, Santiago 8370993, Chile; m.alarconrivera@outlook.cl; 4 Departamento de Gestión de la Información, Universidad Tecnológica Metropolitana, Santiago 7550000, Chile; cflores@utem.cl

**Keywords:** metabolic syndrome, exercise therapy, treatment outcome, insulin resistance, inflammation

## Abstract

Metabolic syndrome (MetS) is a clinical condition defined by the coexistence of interconnected cardiometabolic risk factors, including central obesity, dyslipidemia, elevated blood pressure, and impaired glucose regulation, which collectively increase the risk of type 2 diabetes mellitus and cardiovascular disease. Beyond these clinical diagnostic features, MetS is characterized by complex pathophysiological alterations involving systemic dysregulation of metabolic signaling across adipose tissue, skeletal muscle, liver, vascular endothelium, and the immune system. Key molecular alterations include impaired insulin receptor substrate (IRS)–Akt signaling, chronic nuclear factor kappa B (NF-κB) activation, mitochondrial dysfunction, and oxidative stress. Physical exercise is recognized as a pleiotropic biomedical intervention capable of restoring metabolic homeostasis through coordinated modulation of intracellular signaling pathways and inter-organ communication. Exercise activates AMP-activated protein kinase (AMPK), enhances peroxisome proliferator-activated receptor gamma coactivator-1 alpha (PGC-1α)-mediated mitochondrial biogenesis, and stimulates nuclear factor erythroid 2-related factor 2 (Nrf2)-dependent antioxidant responses. These adaptations improve glucose uptake, enhance fatty acid oxidation, and reduce ectopic lipid accumulation across metabolically active tissues. At the systemic level, skeletal muscle functions as an endocrine organ by releasing myokines such as irisin, interleukin-6 (IL-6), and fibroblast growth factor 21 (FGF21), which contribute to metabolic regulation across the liver, adipose tissue, and vasculature. These exercise-induced signals promote immune modulation, reduce pro-inflammatory cytokine production, and improve endothelial function. Different exercise modalities including aerobic, resistance, and high-intensity interval training (HIIT) activate both common and modality-specific molecular pathways, supporting individualized exercise strategies. Collectively, exercise targets the multi-organ pathophysiology of MetS and provides a mechanistic foundation for precision exercise medicine in cardiometabolic disease management.

## 1. Introduction

Metabolic syndrome (MetS) is a clinical condition defined by the coexistence of interconnected cardiometabolic risk factors, including central obesity, elevated blood pressure, dyslipidemia, and impaired glucose regulation, which collectively increase the risk of cardiovascular disease and type 2 diabetes mellitus worldwide [1,2,3,4]. Beyond its clinical definition, MetS is characterized by a complex pathophysiological network involving dysregulated signaling across multiple organ systems, including adipose tissue, skeletal muscle, liver, vascular endothelium, and immune cells [5,6,7,8]. Although insulin resistance and chronic low-grade inflammation are considered central mechanisms underlying MetS, they are not diagnostic criteria and may not be present in all affected individuals. From a biomedical perspective, MetS is therefore not merely a clinical aggregation of risk factors but a complex network disease involving dysregulated signaling across multiple organ systems, including adipose tissue, skeletal muscle, liver, vascular endothelium, and immune cells [5,6,7,8].

At the cellular level, MetS is driven by persistent metabolic overload and impaired nutrient sensing, leading to disruption of insulin receptor substrate (IRS)–Akt signaling, chronic activation of stress pathways such as c-Jun N-terminal kinase (JNK) and nuclear factor kappa B (NF-κB), and progressive mitochondrial dysfunction [9,10,11,12]. These alterations result in reduced glucose uptake, enhanced hepatic gluconeogenesis, increased lipolysis in adipose tissue, and ectopic lipid deposition in liver and muscle, thereby amplifying systemic metabolic dysfunction [7,13].

A key biomedical hallmark of MetS is chronic low-grade inflammation, characterized by increased circulating levels of tumor necrosis factor-alpha (TNF-α), interleukin-6 (IL-6), and C-reactive protein (CRP), together with altered adipokine profiles including decreased adiponectin and increased leptin [14,15]. This inflammatory milieu contributes to endothelial dysfunction, impaired nitric oxide bioavailability, and accelerated atherogenesis, linking metabolic dysfunction to cardiovascular disease [16,17].

Mitochondrial dysfunction and oxidative stress further exacerbate disease progression by impairing oxidative phosphorylation, increasing reactive oxygen species (ROS) production, and activating redox-sensitive signaling pathways [18,19]. Collectively, these processes create a self-perpetuating cycle of metabolic inflexibility, inflammation, and cellular injury that defines the pathophysiology of MetS.

In this context, physical exercise has emerged as a powerful biomedical intervention capable of modulating multiple disease-driving mechanisms simultaneously [20,21,22,23,24]. Exercise activates AMP-activated protein kinase (AMPK), enhances peroxisome proliferator-activated receptor gamma coactivator-1 alpha (PGC-1α)-mediated mitochondrial biogenesis, improves insulin signaling through IRS–Akt pathway restoration, and activates nuclear factor erythroid 2-related factor 2 (Nrf2)-dependent antioxidant defense systems [25,26,27,28]. Importantly, skeletal muscle acts as an endocrine organ during contraction, releasing myokines such as irisin, IL-6, and fibroblast growth factor 21 (FGF21), which are associated with inter-organ communication and systemic metabolic adaptation [29,30].

Despite substantial advances in understanding the physiological effects of exercise, the integration of its molecular mechanisms into a unified biomedical framework for MetS remains incomplete. A systems-level synthesis linking intracellular signaling, organ crosstalk, and clinical outcomes is required to support its implementation as a precision therapeutic strategy. Therefore, this narrative review aims to critically analyze the molecular and cellular mechanisms through which exercise modulates MetS and to discuss their implications for translational and clinical medicine.

## 2. Literature Search Strategy

Although this manuscript is presented as a narrative review, a structured literature search was performed to ensure a comprehensive and balanced synthesis of current evidence regarding the molecular mechanisms through which exercise modulates MetS. Electronic searches were conducted in PubMed/MEDLINE, Scopus, and Web of Science for articles published from January 2000 to June 2026. The search strategy combined Medical Subject Headings (MeSH) and free-text terms related to exercise and metabolic syndrome, including “*metabolic syndrome*,” “*exercise*,” “*physical activity*,” “*aerobic exercise*,” “*resistance training*,” “*high-intensity interval training*,” “*myokines*,” “*exerkines*,” “*AMPK*,” “*PGC-1α*,” “*Nrf2*,” “*mitochondrial biogenesis*,” “*oxidative stress*,” “*inflammation*,” “*insulin resistance*,” “*organ crosstalk*,” and “*precision exercise*”. Priority was given to systematic reviews, meta-analyses, randomized controlled trials, prospective cohort studies, and high-quality experimental studies published in peer-reviewed journals. Seminal studies that established key molecular mechanisms were also included when considered essential for understanding current concepts. Additional relevant articles were identified through manual screening of the reference lists of selected publications. Research was selected based on its scientific quality, methodological rigor, biological relevance, and direct contribution to understanding the molecular pathways, inter-organ communication, biomarkers, and clinical implications of exercise in MetS.

## 3. Pathophysiological Basis of Metabolic Syndrome: An Integrated Cellular and Molecular Perspective

**Figure 1:**
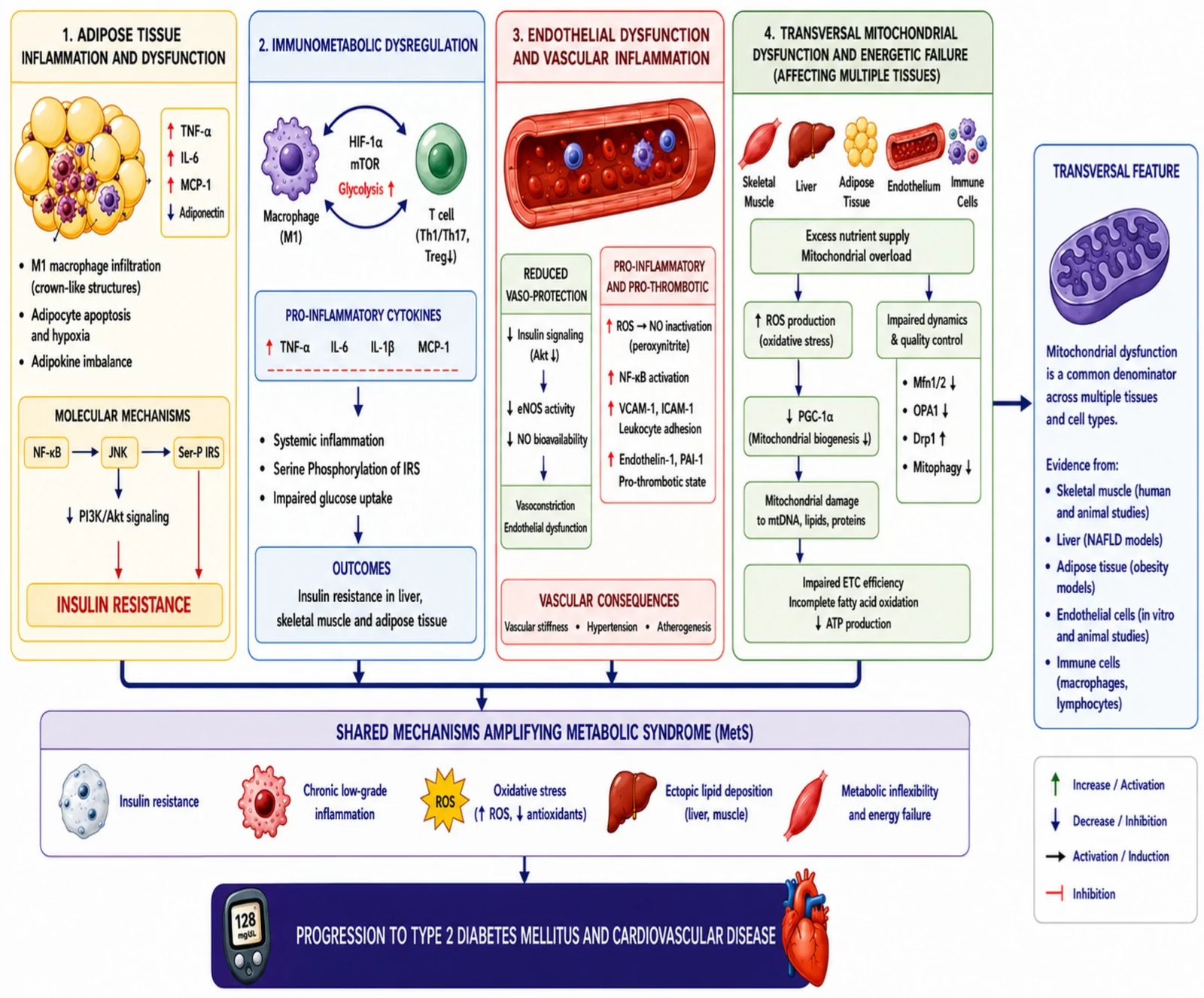
Pathophysiological Background of MetS. Source: own elaboration. Akt: protein kinase B, ETC: electron transport chain, eNOS: endothelial nitric oxide synthase, HIF-1α: hypoxia-inducible factor-1 alpha, ICAM-1: intercellular adhesion molecule-1, IL-1β: interleukin-1 beta, IL-6: interleukin-6, IRS: insulin receptor substrate, JNK: c-Jun N-terminal kinase, MCP-1: monocyte chemoattractant protein-1, MetS: metabolic syndrome, mTOR: mechanistic target of rapamycin, mtDNA: mitochondrial DNA, NF-κB: nuclear factor kappa B, NLRP3: NOD-like receptor family pyrin domain-containing 3, NO: nitric oxide, PGC-1α: peroxisome proliferator-activated receptor gamma coactivator-1 alpha, PI3K: phosphoinositide 3-kinase, ROS: reactive oxygen species, Ser-IRS: serine phosphorylation of insulin receptor substrate, Treg: regulatory T cell, Th1: T helper 1 cell, Th17: T helper 17 cell, TNF-α: tumor necrosis factor-alpha, VCAM-1: vascular cell adhesion molecule-1.

MetS is increasingly recognized as a systemic disorder of inter-organ metabolic dysregulation rather than a collection of isolated clinical risk factors [1]. At the cellular level, its pathophysiology arises from the convergence of adipose tissue dysfunction, immune–metabolic reprogramming, endothelial impairment, and mitochondrial failure [1,12]. These interconnected processes establish a self-sustaining network of metabolic inflexibility, chronic inflammation, and oxidative stress that drives disease progression toward type 2 diabetes mellitus and cardiovascular disease [1,12,13,14] (Figure 1).

### 3.1. Adipose Tissue Inflammation and Dysfunction

Adipose tissue is no longer considered a passive energy storage depot but rather a highly active endocrine and immunometabolic organ that plays a central role in whole-body energy homeostasis and inflammatory regulation [31]. In MetS, chronic positive energy balance drives sustained lipid accumulation within adipocytes, leading initially to hypertrophy and, subsequently, hyperplasia as a compensatory mechanism [32,33]. However, this expansion often exceeds the capacity of the local vascular network, resulting in impaired angiogenesis, reduced oxygen diffusion, and the development of a hypoxic microenvironment within adipose depots [34]. Hypoxia-inducible factor-1α (HIF-1α) is consequently stabilized, triggering transcriptional programs associated with fibrosis, cellular stress, and inflammatory signaling [35,36].

This hypoxic and metabolically stressed environment promotes adipocyte dysfunction, endoplasmic reticulum stress, and eventual apoptosis or necrosis of enlarged adipocytes [37,38]. These cellular events act as chemotactic signals for immune cell infiltration, particularly monocytes that differentiate into classically activated M1-polarized macrophages [39]. These macrophages characteristically aggregate around dead or dying adipocytes, forming crown-like structures (CLS), which are a histological hallmark of adipose tissue inflammation in obesity and MetS [40,41]. The progressive accumulation of CLS reflects a shift from a homeostatic tissue-resident macrophage phenotype toward a sustained pro-inflammatory immune state [42].

Activated macrophages, together with hypertrophic and dysfunctional adipocytes, establish a dysregulated secretory profile characterized by elevated production of pro-inflammatory cytokines and chemokines, including TNF-α, IL-6, and monocyte chemoattractant protein-1 (MCP-1), among others [43,44]. In parallel, there is a marked reduction in the secretion of adiponectin, an anti-inflammatory and insulin-sensitizing adipokine that plays a protective role in glucose and lipid metabolism [45]. This imbalance in adipokine secretion contributes to systemic metabolic dysregulation, promoting insulin resistance in peripheral tissues and favoring ectopic lipid accumulation in organs such as the liver and skeletal muscle, thereby exacerbating lipotoxicity and metabolic impairment [46,47].

At the intracellular signaling level, adipose tissue inflammation is primarily mediated through the activation of canonical inflammatory pathways, including NF-κB and JNK [48]. Activation of these pathways is closely linked to stress responses induced by free fatty acids, oxidative stress, and cytokine signaling [49]. NF-κB and JNK signaling converge to impair insulin receptor substrate (IRS) function through serine phosphorylation, which inhibits its ability to effectively transduce insulin signals [50,51]. This disruption leads to downstream attenuation of the phosphoinositide 3-kinase (PI3K)–Akt signaling cascade, ultimately reducing glucose transporter type 4 (GLUT4) translocation and impairing insulin-stimulated glucose uptake [52].

Collectively, these molecular and cellular alterations establish adipose tissue as a central driver of systemic insulin resistance and chronic low-grade inflammation [53,54]. Beyond its metabolic role, inflamed adipose tissue acts as an active signaling hub that perpetuates crosstalk with the liver, skeletal muscle, and vascular endothelium, thereby contributing to the multisystem pathophysiology characteristic of MetS [55].

### 3.2. Immunometabolic Dysregulation

MetS is fundamentally characterized by immunometabolic reprogramming, where immune cells undergo phenotypic and metabolic shifts that reinforce systemic inflammation [56]. Macrophages in insulin-resistant adipose tissue switch from oxidative phosphorylation to glycolysis-dominant metabolism, a process regulated by hypoxia-inducible factor 1-alpha (HIF-1α) and mechanistic target of rapamycin (mTOR) signaling [57].

This metabolic shift enhances pro-inflammatory cytokine production and sustains chronic inflammation. In parallel, T-cell polarization is altered, with increased Th1 and Th17 responses and reduced regulatory T-cell (Treg) activity, further amplifying inflammatory signaling [58,59]. These immune alterations extend beyond adipose tissue, affecting hepatic Kupffer cells and vascular immune populations [60].

At systemic level, circulating cytokines such as TNF-α and IL-6 induce insulin resistance in liver and skeletal muscle by serine phosphorylation of IRS proteins and suppression of insulin-mediated glucose uptake [61]. This establishes a bidirectional loop between metabolic dysfunction and immune activation, defining MetS as a true immunometabolic disease [62].

Mitochondrial dysfunction is a unifying mechanism underlying metabolic inflexibility in MetS [12]. Excess nutrient supply leads to mitochondrial overload, impaired electron transport chain efficiency, and incomplete fatty acid oxidation [63]. This results in excessive ROS generation, which damages mitochondrial DNA, lipids, and proteins, further impairing oxidative phosphorylation [64,65].

A key defect involves reduced activity of PGC-1α, a master regulator of mitochondrial biogenesis [66]. Downregulation of PGC-1α limits mitochondrial turnover and reduces oxidative capacity in skeletal muscle, liver, and adipose tissue [53,54]. Additionally, impaired mitochondrial dynamics—imbalanced fusion and fission processes mediated by mitofusins (Mfn1/2) and dynamin-related protein 1 (Drp1)—contributes to accumulation of dysfunctional mitochondria [67]. Defective mitophagy further exacerbates cellular stress, leading to activation of inflammasomes such as NLRP3, linking mitochondrial damage to immune activation [68,69].

This energetic failure reinforces insulin resistance, lipid accumulation, and inflammatory signaling, positioning mitochondrial dysfunction as a central hub in MetS pathophysiology [70,71].

### 3.3. Endothelial Dysfunction and Vascular Inflammation

Endothelial dysfunction is a central pathological feature linking metabolic abnormalities to cardiovascular complications in MetS, acting as an early and critical event in the progression toward atherosclerosis and vascular disease [72,73]. Under physiological conditions, insulin exerts vasoprotective effects on the endothelium by activating the phosphoinositide 3-kinase (PI3K)–Akt signaling pathway, which leads to phosphorylation and activation of endothelial nitric oxide synthase (eNOS) [74,75]. This process promotes the production of nitric oxide (NO), a key vasodilatory molecule that maintains vascular tone, inhibits platelet aggregation, and suppresses leukocyte adhesion to the endothelium [76,77]. In addition, NO plays an essential role in preserving endothelial integrity and preventing vascular smooth muscle proliferation [78].

In MetS, insulin resistance disrupts this signaling axis, selectively impairing the PI3K–Akt–eNOS pathway while leaving mitogen-activated protein kinase (MAPK)-dependent vasoconstrictive pathways relatively unopposed [79,80]. As a result, eNOS activity is reduced, leading to decrease NO bioavailability and a shift toward increased vasoconstriction, vascular reactivity, and impaired endothelial-dependent relaxation [74,75,76,77]. This imbalance contributes to early functional alterations in the vasculature, even before structural changes become evident.

Concurrently, metabolic disturbances associated with MetS, including hyperglycemia, elevated free fatty acids, and dyslipidemia, promote excessive production of ROS through mitochondrial dysfunction and activation of NADPH oxidase [18,81]. These ROS rapidly inactivate NO by forming peroxynitrite, a highly reactive nitrogen species that not only reduces NO bioavailability but also induces oxidative damage to lipids, proteins, and DNA within endothelial cells [82]. This oxidative stress amplifies endothelial injury and further perpetuates vascular dysfunction in a self-sustaining loop [83].

At the inflammatory signaling level, activation of NF-κB in endothelial cells plays a pivotal role in propagating vascular inflammation [84]. NF-κB upregulates the expression of adhesion molecules, including vascular cell adhesion molecule-1 (VCAM-1) and intercellular adhesion molecule-1 (ICAM-1), which facilitate the adhesion, rolling, and transmigration of circulating leukocytes into the subendothelial space [85,86]. This process marks a key step in the initiation and progression of atherosclerotic plaque formation, promoting chronic vascular wall inflammation and immune cell infiltration [87].

In parallel, endothelial cells undergo a phenotypic shift toward a pro-thrombotic and pro-constrictive state [1]. This is characterized by increased expression of endothelin-1, a potent vasoconstrictor peptide, and plasminogen activator inhibitor-1 (PAI-1), which inhibits fibrinolysis and promotes thrombus stability [88,89]. These changes contribute to increased vascular stiffness, elevated peripheral resistance, hypertension, and accelerated atherogenesis [89]. Over time, the combined effects of impaired vasodilation, oxidative stress, inflammation, and pro-thrombotic signaling establish endothelial dysfunction as a key vascular manifestation of systemic metabolic inflammation in MetS, bridging metabolic dysregulation with overt cardiovascular disease [88].

### 3.4. Mitochondrial Dysfunction and Cellular Energetic Failure

Mitochondrial dysfunction is widely recognized as a unifying mechanistic hallmark underlying metabolic inflexibility in MetS, integrating disturbances in energy metabolism, redox balance, and inflammatory signaling [12]. Under conditions of chronic nutrient excess, particularly elevated glucose and free fatty acid availability, mitochondria become metabolically overloaded, leading to saturation of oxidative phosphorylation capacity [63,90]. This overload disrupts the normal coupling efficiency of the electron transport chain (ETC), resulting in electron leakage at complexes I and III and impaired proton gradient maintenance [90,91,92]. As a consequence, incomplete β-oxidation of fatty acids occurs, favoring the accumulation of lipid intermediates such as diacylglycerols and ceramides, which further interfere with insulin signaling pathways [93].

A major downstream consequence of ETC inefficiency is the excessive generation of ROS, which overwhelms endogenous antioxidant defenses such as superoxide dismutase and glutathione peroxidase [94,95]. Elevated ROS levels induce oxidative damage to mitochondrial DNA (mtDNA), which is particularly vulnerable due to its proximity to the ETC and limited repair capacity [96,97]. In addition, ROS-mediated lipid peroxidation compromises mitochondrial membrane integrity, while protein oxidation alters the function of key respiratory chain complexes, further exacerbating mitochondrial inefficiency and establishing a self-perpetuating cycle of energetic dysfunction [96,97].

A critical molecular defect in MetS involves the downregulation and functional impairment of PGC-1α, a master transcriptional coactivator regulating mitochondrial biogenesis and oxidative metabolism [66,98,99]. Reduced PGC-1α activity limits the transcription of nuclear and mitochondrial genes required for ETC assembly, fatty acid oxidation, and mitochondrial replication [100]. This impairment leads to diminished mitochondrial density and reduced oxidative capacity in metabolically active tissues, including skeletal muscle, liver, and adipose tissue, thereby contributing to systemic metabolic inflexibility and reduced substrate switching between lipids and carbohydrates [53,54].

In addition to defects in mitochondrial biogenesis, MetS is characterized by profound alterations in mitochondrial dynamics, particularly the balance between fusion and fission processes [101,102]. Mitochondrial fusion, mediated by mitofusins (Mfn1 and Mfn2) and optic atrophy 1 (OPA1), is essential for maintaining mitochondrial integrity and functional complementation, whereas fission, regulated primarily by dynamin-related protein 1 (Drp1), facilitates mitochondrial quality control and removal of damaged organelles [67,103,104,105]. In MetS, this balance is disrupted in favor of excessive fission and/or impaired fusion, leading to fragmented and energetically inefficient mitochondria [106]. Such structural abnormalities compromise ATP production and increase susceptibility to oxidative stress [107].

Furthermore, defective mitophagy, the selective autophagic removal of damaged mitochondria, prevents the efficient clearance of dysfunctional organelles, allowing their accumulation within cells [108]. This failure of mitochondrial quality control amplifies cellular stress signaling and contributes to the activation of innate immune pathways, particularly the NOD-like receptor family pyrin domain-containing 3 (NLRP3) inflammasome [109,110]. Activation of the NLRP3 inflammasome links mitochondrial dysfunction directly to chronic low-grade inflammation through the maturation and release of pro-inflammatory cytokines such as interleukin-1β (IL-1β) and interleukin-18 (IL-18) [109,110,111].

Collectively, these interrelated mechanisms establish mitochondrial dysfunction as a central hub in the pathophysiology of MetS, bridging impaired energy metabolism with oxidative stress, insulin resistance, lipid accumulation, and chronic inflammation [12]. This energetic failure not only compromises cellular homeostasis but also reinforces systemic metabolic dysregulation, positioning mitochondria as a key integrative node in disease progression [12].

### 3.5. Inter-Organ Communication and Exercise-Induced Crosstalk in MetS

MetS is increasingly recognized as a disorder of impaired inter-organ communication rather than a condition confined to individual tissues [1,55,112]. Under physiological conditions, skeletal muscle, adipose tissue, liver, vascular endothelium, and immune cells continuously exchange endocrine, paracrine, and autocrine signals that coordinate energy metabolism and maintain metabolic homeostasis [1,55,112]. In MetS, this communication network becomes profoundly disrupted, leading to maladaptive signaling loops that perpetuate insulin resistance, chronic inflammation, and metabolic inflexibility [1,55,112].

Adipose tissue plays a central role in this dysregulated crosstalk through the excessive secretion of pro-inflammatory adipokines and cytokines, including TNF-α, IL-6, and MCP-1, while simultaneously reducing the release of protective mediators such as adiponectin [113,114]. These alterations promote insulin resistance in skeletal muscle and liver, impair endothelial function, and facilitate immune cell activation [115]. In parallel, hepatic lipid accumulation contributes to the release of hepatokines such as fetuin-A, which further suppress insulin signaling and amplify systemic inflammation [116].

Skeletal muscle represents a major regulatory hub within this communication network [117]. Beyond its role in glucose disposal, contracting skeletal muscle functions as an endocrine organ through the release of myokines, including irisin, IL-6, FGF21, and brain-derived neurotrophic factor (BDNF), which have been implicated in exercise-induced metabolic adaptation [118,119]. These molecules exert systemic effects on adipose tissue metabolism, hepatic lipid handling, endothelial function, and immune regulation [120]. Exercise-induced myokine secretion promotes browning of white adipose tissue, enhances fatty acid oxidation, improves insulin sensitivity, and attenuates chronic inflammatory signaling, thereby counteracting many of the pathophysiological mechanisms underlying MetS [21,121,122].

The immune system acts as both a mediator and amplifier of inter-organ communication [123]. Pro-inflammatory macrophages and activated T lymphocytes produce cytokines that influence insulin signaling in multiple tissues, while metabolic stress-derived signals from adipocytes and hepatocytes further modulate immune cell phenotype and function [124,125]. This bidirectional communication establishes a self-reinforcing cycle between metabolic dysfunction and chronic inflammation that contributes to disease progression [126].

Exercise represents one of the most effective interventions for restoring physiological organ crosstalk [127,128]. Through coordinated modulation of myokines, adipokines, hepatokines, endothelial mediators, and immune signaling pathways, exercise re-establishes communication between metabolic organs and promotes systemic homeostasis [127,128]. Consequently, the therapeutic effects of exercise in MetS should not be viewed solely as tissue-specific adaptations but rather as the restoration of an integrated network of inter-organ signaling that underlies cardiometabolic health [21,122].

## 4. Exercise as a Molecular Modulator of MetS

**Figure 2:**
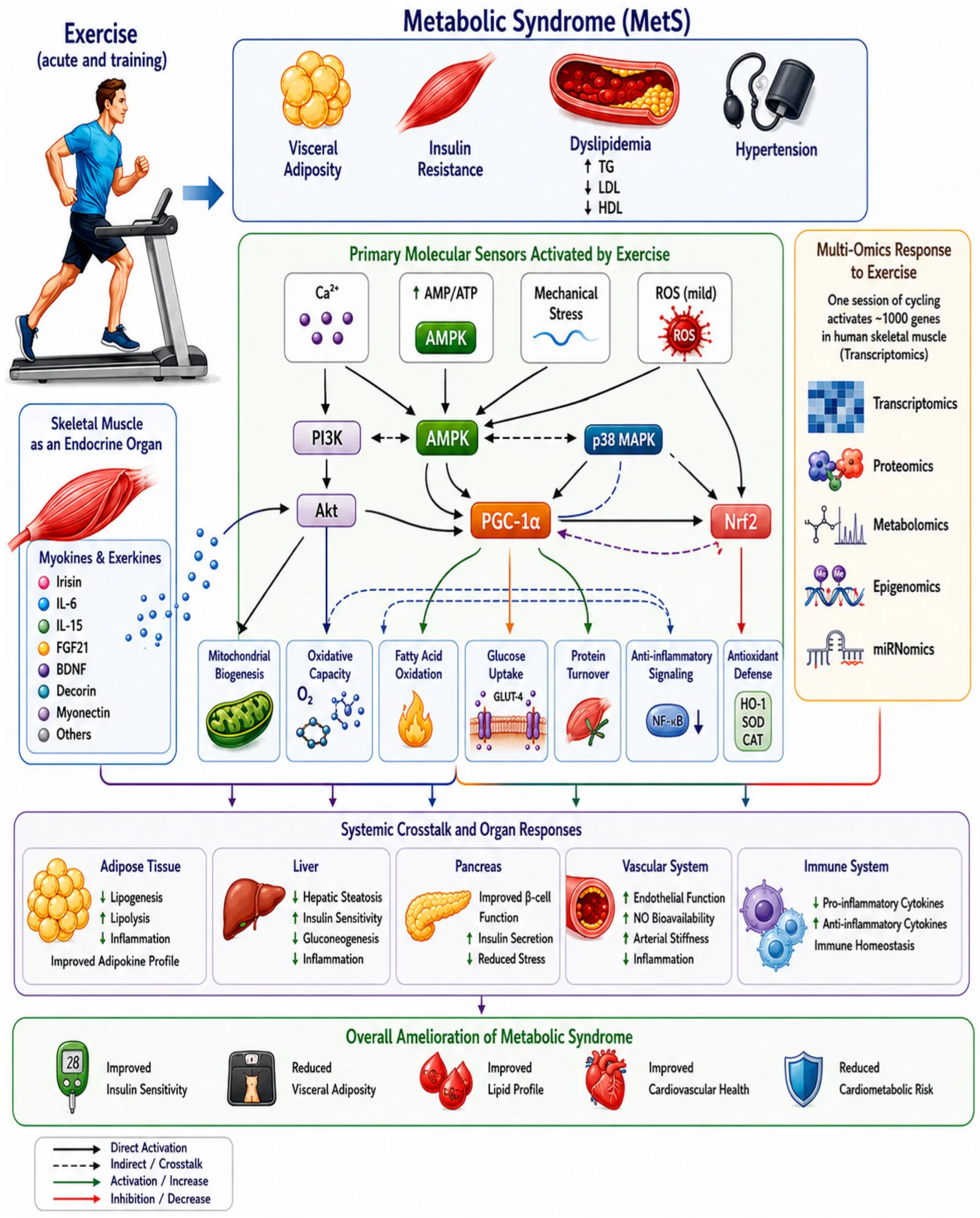
Exercise as a Molecular Modulator of Metabolic Syndrome. Source: own elaboration. Akt: protein kinase B, AMPK: AMP: activated protein kinase, BDNF: brain-derived neurotrophic factor, CaMKII: calcium/calmodulin-dependent protein kinase II, CAT: catalase, ETC: electron transport chain, FGF21: fibroblast growth factor 21, GLUT4: glucose transporter type 4, HDL: high-density lipoprotein cholesterol, HO-1: heme oxygenase-1, IL-6: interleukin-6, IL-15: interleukin-15, LDL: low-density lipoprotein cholesterol, MAPK: mitogen-activated protein kinase, MetS: metabolic syndrome, mtDNA: mitochondrial DNA, NF-κB: nuclear factor kappa B, Nrf2: nuclear factor erythroid 2-related factor 2, PGC-1α: peroxisome proliferator-activated receptor gamma coactivator 1-alpha, PI3K: phosphoinositide 3-kinase, ROS: reactive oxygen species, SOD: superoxide dismutase, TG: triglycerides.

Regular exercise activates key signaling pathways, including AMPK, PGC-1α, PI3K/Akt, and Nrf2, resulting in improved insulin sensitivity, enhanced mitochondrial function, increased fatty acid oxidation, improved glucose uptake, and attenuation of chronic inflammation and oxidative stress. Furthermore, skeletal muscle functions as an endocrine organ by releasing myokines such as irisin, IL-6, and FGF21, which have been implicated in inter-organ communication with adipose tissue, the liver, and other metabolic organs, thereby contributing to systemic metabolic homeostasis (Figure 2).

### 4.1. Regulation of Glucose and Lipid Metabolism

Physical exercise is a cornerstone non-pharmacological intervention for the prevention and management of MetS, targeting multiple interconnected mechanisms underlying insulin resistance, dyslipidemia, visceral adiposity, and impaired metabolic flexibility [21,23,24,121,129].

From a translational perspective, exercise should no longer be regarded merely as a lifestyle recommendation but rather as a dose-dependent intervention that induces systemic molecular adaptations with multi-target effects [130,131]. Similar to pharmacological interventions, its therapeutic efficacy depends on exercise modality, intensity, frequency, and duration, each of which activates distinct but interconnected signaling pathways [132]. Unlike conventional drugs that generally target a single molecular pathway, exercise simultaneously influences skeletal muscle, adipose tissue, liver, vasculature, and immune function, thereby addressing the multisystem nature of MetS [21,23,24,121,129].

One of the primary molecular effects of exercise in MetS is the regulation of glucose and lipid metabolism [133]. Contractile activity increases cellular energy demand, leading to activation of AMP-activated protein kinase (AMPK), a central energy sensor that promotes catabolic pathways while suppressing anabolic processes [25]. AMPK activation enhances fatty acid oxidation through inhibition of acetyl-CoA carboxylase, decreases malonyl-CoA concentrations, and facilitates mitochondrial fatty acid transport and utilization [25,134]. Simultaneously, exercise suppresses lipogenesis and improves lipid utilization, thereby reducing ectopic fat accumulation and circulating triglyceride levels [135].

A major downstream effector of AMPK is PGC-1α, which promotes oxidative metabolism and metabolic flexibility [136]. Through activation of the AMPK–PGC-1α axis, exercise enhances mitochondrial oxidative capacity, improves substrate utilization, and facilitates the transition from lipid storage toward lipid oxidation [137]. These adaptations are particularly relevant in MetS, where impaired metabolic flexibility contributes to chronic energy surplus and insulin resistance [122].

Exercise also enhances insulin signaling through activation of the phosphoinositide 3-kinase/protein kinase B (PI3K/Akt) pathway [27,122]. This signaling cascade promotes glucose transporter type 4 (GLUT4) translocation to the plasma membrane, increasing glucose uptake in skeletal muscle and adipose tissue [138]. Restoration of PI3K/Akt signaling improves insulin-mediated glucose disposal and contributes to reductions in insulin resistance and hyperglycemia [27].

Different exercise modalities induce complementary metabolic adaptations [139]. Aerobic exercise predominantly enhances oxidative metabolism through sustained AMPK activation and increased PGC-1α signaling, while also promoting angiogenesis through vascular endothelial growth factor (VEGF), thereby improving oxygen delivery and substrate utilization [140,141]. Resistance training induces mechanical tension and muscle fiber recruitment, activating mTOR pathways that promote protein synthesis, muscle remodeling, and increases in lean body mass [142]. Given that skeletal muscle is the primary site of postprandial glucose disposal, these adaptations play a critical role in improving glycemic control and insulin sensitivity [139]. High-Intensity Interval Training (HIIT) integrates metabolic and mechanical stressors, producing robust activation of AMPK, calcium/calmodulin-dependent protein kinase (CaMK), and p38 mitogen-activated protein kinase (p38 MAPK), thereby enhancing mitochondrial biogenesis, glucose transport capacity, and cardiorespiratory fitness despite relatively low training volumes [143,144,145].

Furthermore, exercise induces favorable remodeling of adipose tissue biology. Reductions in visceral adiposity, improvements in adipocyte function, increased adiponectin secretion, and attenuation of leptin resistance contribute to enhanced insulin sensitivity and systemic metabolic regulation [137]. Collectively, these integrated adaptations directly target the central metabolic disturbances that characterize MetS [23].

### 4.2. Mitochondrial Adaptations Induced by Exercise

Exercise-induced improvements in mitochondrial function represent a fundamental mechanism through which physical activity counteracts MetS [23]. Mitochondrial dysfunction is increasingly recognized as a central contributor to insulin resistance, impaired substrate oxidation, reduced metabolic flexibility, and cardiometabolic disease progression [146,147].

Regular exercise stimulates the expression and activation of PGC-1α, a master regulator of mitochondrial biogenesis and oxidative metabolism [99]. Activation of PGC-1α occurs through multiple upstream pathways, including AMPK, CaMK, and p38 MAPK, which respond to energetic and mechanical stress generated during exercise [66,99]. Upregulation of PGC-1α promotes coordinated transcription of genes involved in mitochondrial respiration, oxidative phosphorylation, ATP synthesis, and fatty acid oxidation, resulting in increased mitochondrial density and improved bioenergetic efficiency [136].

Enhanced mitochondrial function increases the capacity for substrate oxidation and reduces the accumulation of toxic lipid intermediates associated with insulin resistance [148,149]. Consequently, exercise restores metabolic flexibility, enabling more efficient switching between carbohydrate and lipid utilization according to metabolic demand [150].

Beyond increasing mitochondrial quantity, exercise also improves mitochondrial quality through modulation of mitochondrial dynamics, including fusion, fission, and mitophagy [103,106,107]. Enhanced turnover of dysfunctional mitochondria preserves cellular bioenergetics and maintains metabolic competence [67]. These adaptations reduce reactive oxygen species production and improve mitochondrial respiratory efficiency [139].

Importantly, the magnitude of mitochondrial adaptation appears to be exercise-modality dependent [132]. Endurance-oriented exercise primarily promotes mitochondrial expansion through sustained activation of AMPK–PGC-1α signaling, whereas HIIT induces comparable increases in mitochondrial biogenesis through repeated energetic perturbations despite lower overall exercise volume [22,140,141,143]. Emerging evidence also suggests considerable inter-individual variability in mitochondrial responsiveness, influenced by baseline fitness, insulin sensitivity, metabolic phenotype, and genetic background [137]. Such findings support the growing concept of personalized exercise interventions aimed at maximizing mitochondrial adaptations in MetS [122].

Through improvements in both mitochondrial quantity and quality, exercise directly addresses one of the central pathophysiological defects underlying MetS, namely impaired oxidative metabolism and reduced metabolic flexibility [122].

### 4.3. Anti-Inflammatory and Antioxidant Effects

Chronic low-grade inflammation and oxidative stress are key drivers of MetS and contribute directly to insulin resistance, endothelial dysfunction, and cardiovascular risk [151,152]. Regular exercise exerts potent anti-inflammatory and antioxidant effects that attenuate these pathological processes and promote metabolic resilience [153].

Physical activity reduces activation of inflammatory signaling pathways such as nuclear factor-kappa B (NF-κB) and c-Jun N-terminal kinase (JNK), resulting in decreased production of pro-inflammatory cytokines including TNF-α, interleukin-1β (IL-1β), and monocyte chemoattractant protein-1 (MCP-1) [154,155,156]. These effects alleviate chronic metabolic inflammation and improve insulin signaling across multiple tissues [137].

Exercise-induced reductions in inflammatory burden are reflected by decreases in circulating biomarkers such as high-sensitivity C-reactive protein (hs-CRP) and TNF-α, both of which are strongly associated with cardiometabolic risk and endothelial dysfunction [157,158]. Improvements in adipose tissue function further contribute to anti-inflammatory effects through increased adiponectin concentrations and improvements in the adiponectin-to-leptin ratio, an emerging marker of adipose tissue health and systemic metabolic regulation [159].

In parallel, exercise activates nuclear factor erythroid 2-related factor 2 (Nrf2), a master regulator of cellular antioxidant defense mechanisms [28]. Nrf2 activation stimulates transcription of antioxidant enzymes including superoxide dismutase (SOD), catalase, and glutathione peroxidase, enhancing endogenous protection against oxidative stress [160]. Consequently, exercise reduces lipid peroxidation, decreases circulating malondialdehyde (MDA) levels, and limits oxidative damage to proteins, lipids, and nucleic acids [161].

The coordinated suppression of inflammatory pathways and enhancement of antioxidant defenses interrupts the self-perpetuating cycle linking oxidative stress, adipose tissue dysfunction, insulin resistance, and cardiometabolic deterioration [21,162,163]. These molecular adaptations not only contribute to improved metabolic health but also provide measurable biomarkers that may be useful for monitoring exercise responsiveness in individuals with MetS [164].

### 4.4. Endocrine Communication and Myokine Signaling

An increasingly recognized mechanism underlying the metabolic benefits of exercise is the endocrine communication between skeletal muscle and other metabolically active tissues [137]. During contraction, skeletal muscle functions as an endocrine organ by releasing a diverse repertoire of myokines, extracellular vesicles, and signaling molecules that coordinate systemic metabolic adaptations [137,165].

Among the growing repertoire of exercise-induced myokines, irisin, IL-6, FGF21, myonectin (CTRP15), meteorin-like (METRNL), brain-derived neurotrophic factor (BDNF), and decorin have been implicated in the regulation of metabolic processes [137,166,167,168]. Irisin, generated by proteolytic cleavage of fibronectin type III domain-containing protein 5 (FNDC5) following exercise-induced activation of PGC-1α, promotes the browning of white adipose tissue through induction of uncoupling protein-1 (UCP1), thereby enhancing mitochondrial thermogenesis, energy expenditure, and systemic insulin sensitivity [137,139]. Exercise-induced IL-6, when released acutely from contracting skeletal muscle, acts as a metabolic regulator rather than a pro-inflammatory cytokine, promoting glucose uptake, lipid mobilization, and anti-inflammatory signaling [169]. Mechanistically, muscle-derived IL-6 activates AMPK in skeletal muscle and liver, stimulating GLUT4 translocation, glucose uptake, and fatty acid oxidation. Concurrently, IL-6 activates the Janus kinase/signal transducer and activator of transcription 3 (JAK/STAT3) pathway in immune cells and hepatocytes, promoting the production of anti-inflammatory mediators such as IL-10 while suppressing TNF-α. Importantly, these transient exercise-induced responses differ fundamentally from the chronic elevation of IL-6 observed in obesity and metabolic syndrome, where persistent signaling contributes to low-grade inflammation and insulin resistance. Similarly, FGF21 contributes to improvements in fatty acid oxidation, mitochondrial function, energy expenditure, and metabolic flexibility [29]. Myonectin promotes fatty acid uptake and lipid homeostasis, METRNL contributes to adipose tissue remodeling and anti-inflammatory responses, BDNF regulates energy balance and glucose metabolism, whereas decorin has been associated with skeletal muscle remodeling and improved insulin sensitivity [166,167,168]. In addition, myostatin acts as a negative regulator of skeletal muscle mass through activation of the activin receptor type IIB (ActRIIB) and the transforming growth factor-beta (TGF-β)/SMAD2/3 signaling pathway. Chronic elevation of myostatin suppresses protein synthesis, impairs mitochondrial function, and contributes to insulin resistance and metabolic dysfunction, whereas regular exercise downregulates myostatin expression, thereby facilitating muscle hypertrophy, metabolic flexibility, and improved glucose homeostasis.

The circulating concentrations of irisin, IL-6, and FGF21 have therefore emerged as potential biomarkers of exercise responsiveness, reflecting the endocrine activity of skeletal muscle and the extent of systemic metabolic remodeling induced by training [170]. In addition to classical myokines, exercise influences adipokines, hepatokines, and immune-derived mediators that collectively participate in inter-organ communication [137].

Beyond protein-based mediators, exercise modulates the release of extracellular vesicles and exercise-responsive microRNAs (miRNAs), including miR-126, miR-133, and miR-206 [171,172,173]. These small non-coding RNAs regulate pathways involved in angiogenesis, myogenesis, mitochondrial biogenesis, inflammation, and metabolic reprogramming while simultaneously acting as mediators of intercellular communication [174]. Specifically, miR-126 promotes endothelial repair and angiogenesis by regulating vascular endothelial growth factor (VEGF)-dependent signaling and the PI3K/Akt/endothelial nitric oxide synthase (eNOS) pathway, thereby improving endothelial function and vascular homeostasis. In contrast, miR-133a and miR-206 regulate skeletal muscle regeneration by modulating satellite cell activation and myogenic differentiation through the regulation of serum response factor (SRF), paired box protein Pax7 (PAX7), and other genes involved in muscle remodeling. Emerging evidence further indicates that exercise-responsive miRNAs regulate key metabolic signaling pathways, including phosphatidylinositol-3 kinase/protein kinase B (PI3K/Akt), mammalian target of rapamycin (mTOR), AMP-activated protein kinase (AMPK), and Forkhead box O (FOXO), thereby influencing protein synthesis, autophagy, mitochondrial adaptation, and insulin sensitivity [137]. Rather than acting independently, these exercise-responsive miRNAs orchestrate coordinated regulatory networks linking specific target genes with intracellular signaling pathways involved in angiogenesis, mitochondrial remodeling, muscle regeneration, inflammation, and metabolic adaptation, providing an additional layer of molecular regulation underlying the systemic benefits of exercise. Due to their stability in circulation and tissue-specific expression patterns, these molecules have emerged as promising biomarkers for monitoring long-term exercise adaptations [137].

Beyond miRNAs, exercise also induces the transient release of circulating cell-free DNA (cfDNA) derived from skeletal muscle, immune cells, and other metabolically active tissues [171,172,173,174]. Although traditionally considered a biomarker of cellular stress, accumulating evidence suggests that cfDNA may participate in intercellular communication by modulating inflammatory responses, immune activation, and metabolic signaling across multiple organs, representing an additional component of the exercise-induced endocrine response. However, its physiological role remains under active investigation [171,172,173,174].

Recent advances in transcriptomics, proteomics, metabolomics, and microbiome research have further expanded the understanding of exercise-induced endocrine signaling networks [128]. Multi-omics approaches reveal coordinated adaptations across skeletal muscle, adipose tissue, liver, vasculature, immune cells, and the gut microbiota, providing a systems-level perspective of exercise biology [137,165]. These technologies have identified molecular signatures associated with improvements in insulin sensitivity, mitochondrial function, inflammatory regulation, and substrate utilization, offering new opportunities for individualized exercise prescription [175].

Through this integrated endocrine network, exercise coordinates metabolic responses across multiple organs, reinforcing systemic insulin sensitivity, metabolic flexibility, and whole-body metabolic homeostasis [137]. This inter-organ crosstalk highlights exercise as a powerful biological regulator capable of addressing the multifactorial nature of MetS and supports the emerging paradigm of precision exercise medicine [123].

## 5. Influence of Exercise Modalities on Molecular Pathways in MetS

**Figure 3:**
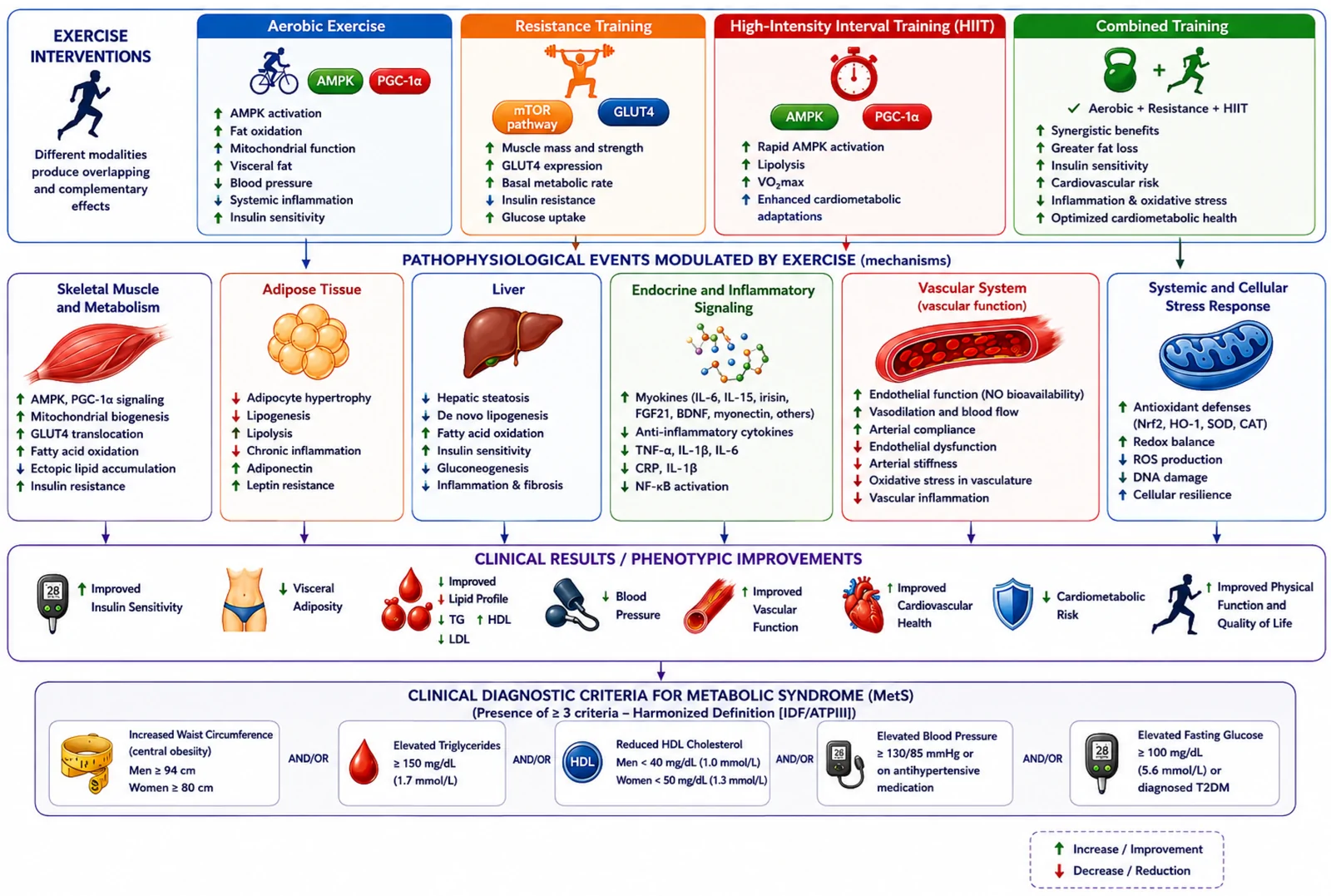
Influence of Exercise Modalities on MetS. Source: own elaboration. AMPK: AMP-activated protein kinase, ATP: adenosine triphosphate, BDNF: brain-derived neurotrophic factor, CAT: catalase, CRP: C-reactive protein, FGF21: fibroblast growth factor 21, GLUT4: glucose transporter type 4, HDL: high-density lipoprotein cholesterol, HIIT: high-intensity interval training, HO-1: heme oxygenase-1, IDF: International Diabetes Federation, IL-1β: interleukin-1 beta, IL-6: interleukin-6, IL-15: interleukin-15, LDL: low-density lipoprotein cholesterol, MetS: metabolic syndrome, mTOR: mechanistic target of rapamycin, NF-κB: nuclear factor kappa B, NO: nitric oxide, Nrf2: nuclear factor erythroid 2-related factor 2, PGC-1α: peroxisome proliferator-activated receptor gamma coactivator-1 alpha, ROS: reactive oxygen species, SOD: superoxide dismutase, T2DM: type 2 diabetes mellitus, TG: triglycerides, TNF-α: tumor necrosis factor-alpha, VO_2_max: maximal oxygen uptake.

The mechanistic pathways underlying MetS provide a strong rationale for exercise as a first-line, multi-target therapeutic strategy [122]. Exercise simultaneously modulates metabolic, inflammatory, vascular, and mitochondrial pathways, positioning it as a systems-level intervention that targets multiple pathophysiological mechanisms underlying MetS [21,122]. Current evidence supporting these molecular adaptations has been obtained predominantly from skeletal muscle, although important contributions from adipose tissue, vascular endothelium, liver, and immune cells have also been reported (Figure 3).

### 5.1. Aerobic Exercise and Oxidative Metabolic Remodeling

Growing evidence indicates that distinct exercise modalities induce partially overlapping yet mechanistically specific molecular adaptations relevant to MetS [21,122]. Aerobic training predominantly enhances oxidative metabolism through sustained activation of AMPK and upregulation of PGC-1α, leading to increased mitochondrial biogenesis, enhanced oxidative phosphorylation, and greater fatty acid oxidation capacity [22,140,141,143].

Prolonged rhythmic muscle contractions increase energy demand and promote systemic lipid mobilization, facilitating the utilization of fatty acids as metabolic substrates [6,117,139]. These adaptations reduce circulating triglyceride levels, decrease visceral adiposity, and improve metabolic flexibility. In parallel, aerobic exercise suppresses lipogenic pathways while enhancing mitochondrial lipid oxidation, thereby improving overall lipid homeostasis [6,117,139].

Aerobic training also promotes angiogenesis through increased VEGF signaling, improving capillary density, oxygen delivery, and nutrient transport in metabolically active tissues [176,177]. Enhanced vascular function contributes to improved insulin sensitivity and mitochondrial efficiency, further supporting cardiometabolic health [176].

Collectively, these adaptations result in improved glucose regulation, reduced adiposity, enhanced insulin sensitivity, and greater oxidative capacity, making aerobic exercise a cornerstone intervention for individuals with MetS [21,122].

### 5.2. Resistance Training and Peripheral Metabolic Control

Although traditionally associated with musculoskeletal adaptations, resistance training has emerged as a potent metabolic intervention capable of improving several components of MetS independently of substantial weight loss [178,179].

By increasing skeletal muscle mass and strength, resistance exercise enhances whole-body glucose disposal and elevates resting metabolic rate [180]. Given that skeletal muscle represents the largest site of insulin-stimulated glucose uptake, increases in lean mass substantially improve glycemic control and insulin sensitivity [181].

At the molecular level, resistance training stimulates PI3K/Akt signaling and promotes GLUT4 translocation to the sarcolemmal membrane, facilitating both insulin-dependent and insulin-independent glucose uptake [182,183]. Concurrent activation of mTOR signaling promotes protein synthesis, muscle remodeling, and hypertrophic adaptations that contribute to long-term metabolic improvements [142].

Beyond glucose regulation, resistance exercise favorably influences adipokine profiles and stimulates the secretion of myokines involved in inflammation control, mitochondrial function, and lipid metabolism [184]. These endocrine adaptations contribute to reductions in insulin resistance and improvements in metabolic flexibility [185].

Consequently, resistance training represents a particularly valuable strategy for individuals with sarcopenic obesity, impaired glucose regulation, or reduced functional capacity, all of which frequently coexist within the MetS phenotype [186].

### 5.3. High-Intensity Interval Training and Rapid Metabolic Adaptations

HIIT combines brief periods of vigorous exercise with recovery intervals, generating substantial metabolic stress within a relatively short time frame [187]. This modality produces rapid perturbations in cellular energy balance that strongly activate AMPK, CaMK, and p38 mitogen-activated protein kinase (p38 MAPK) [143,145].

The convergence of these signaling pathways results in robust stimulation of PGC-1α, promoting mitochondrial biogenesis, oxidative enzyme activity, and improvements in metabolic flexibility [188]. HIIT has consistently demonstrated the ability to enhance insulin sensitivity, increase glucose transport capacity, and improve cardiorespiratory fitness despite lower total exercise volume compared with traditional endurance training [189,190]. The high-intensity nature of HIIT also increases catecholamine-mediated lipolysis, facilitating mobilization of fatty acids from adipose tissue and contributing to reductions in visceral fat mass [191]. Furthermore, HIIT has been associated with significant reductions in inflammatory biomarkers and improvements in endothelial function, making it an attractive time-efficient strategy for individuals with MetS [22].

Given its ability to simultaneously target mitochondrial dysfunction, insulin resistance, adiposity, and cardiovascular fitness, HIIT represents one of the most effective exercise modalities for inducing rapid cardiometabolic adaptations [192].

### 5.4. Combined Training Approaches and Integrated Molecular Benefits

Although aerobic, resistance, and HIIT modalities activate distinct primary pathways, all forms of exercise ultimately converge on common molecular targets relevant to MetS, including activation of AMPK and PGC-1α signaling, enhancement of insulin responsiveness through PI3K/Akt pathways, suppression of chronic inflammatory signaling, stimulation of antioxidant defenses through Nrf2 activation, and improvements in mitochondrial quality and function [188,193,194].

Importantly, many exercise-induced metabolic benefits occur independently of substantial weight loss, emphasizing that exercise exerts direct molecular and cellular effects beyond simple reductions in body mass [195]. Improvements in insulin sensitivity, inflammatory status, mitochondrial function, and metabolic flexibility can therefore occur even when changes in body weight are modest [196].

Emerging evidence suggests that combined training approaches integrating aerobic and resistance exercise may provide additive or synergistic benefits by simultaneously targeting oxidative capacity, muscle mass, glucose regulation, and cardiometabolic fitness [157]. Such multimodal programs appear particularly effective for reducing visceral adiposity, improving glycemic control, attenuating systemic inflammation, and enhancing physical function [195].

From a precision exercise medicine perspective, exercise prescription should be individualized according to metabolic phenotype, functional capacity, comorbidities, and patient preferences [197]. Individuals with predominant visceral obesity and insulin resistance may benefit from greater emphasis on aerobic or HIIT interventions, whereas those with sarcopenia or reduced functional capacity may require a stronger resistance training component [198,199]. Consequently, combined and periodized exercise programs may represent the most comprehensive strategy for optimizing molecular, physiological, and clinical outcomes in MetS [200].

## 6. Clinical and Translational Implications of Exercise in MetS

The expanding understanding of exercise-induced molecular adaptations provides a compelling framework for translating mechanistic insights into clinical strategies for metabolic syndrome management [137]. By simultaneously targeting insulin resistance, adipose tissue dysfunction, mitochondrial impairment, chronic low-grade inflammation, and inter-organ communication, exercise addresses the multifactorial nature of metabolic syndrome more comprehensively than most pharmacological approaches [201]. These pleiotropic effects support the integration of structured physical activity as a cornerstone intervention across the entire cardiometabolic disease spectrum, from early metabolic dysfunction to advanced cardiovascular risk states [202,203].

Importantly, exercise-mediated improvements in insulin sensitivity, glycemic control, lipid metabolism, blood pressure regulation, and body composition frequently occur independently of substantial weight loss, emphasizing that metabolic remodeling rather than body mass reduction per se drives many of the health benefits associated with physical activity [204]. This observation has considerable clinical relevance, as it broadens therapeutic opportunities for individuals who struggle to achieve or maintain meaningful weight loss [205]. Moreover, improvements in cardiorespiratory fitness and muscle strength provide additional protection against cardiovascular morbidity and mortality [137,206].

From a translational perspective, emerging biomarkers reflecting exercise responsiveness may facilitate individualized intervention strategies [164]. Circulating levels of FGF21, irisin, inflammatory cytokines, adipokines, oxidative stress markers, and exercise-regulated microRNAs may provide valuable insights into metabolic adaptations to exercise. Nevertheless, their clinical utility for personalized exercise prescription has not yet been established and remains an area of ongoing investigation [29,169,170,171,172,173,174,175]. Advanced imaging modalities assessing body composition and visceral adiposity, along with non-invasive metabolic assessments such as glucose homeostasis indices, lipid profiles, and vascular function measures, further enable monitoring of physiological responses to training [21,23,24,121,129,137,191,195]. Integration of these biomarkers into clinical practice may allow stratification of patients according to molecular phenotype and predicted responsiveness to specific exercise modalities [164,170].

The heterogeneity of MetS underscores the need for personalized exercise prescriptions [20,21,22,23,24]. Patients differ markedly in metabolic status, degree of insulin resistance, adiposity distribution, physical capacity, and comorbidity burden, necessitating tailored programs that balance efficacy, adherence, and safety [20,21,22,23,24]. Aerobic exercise may be prioritized in individuals with obesity, dyslipidemia, and impaired cardiorespiratory fitness, whereas resistance training may provide particular benefits in sarcopenic, insulin-resistant, or physically deconditioned phenotypes [21,122,176,177]. HIIT offers a time-efficient alternative for selected individuals, while combined aerobic and resistance training may maximize cardiometabolic adaptations and overall health outcomes [143,145,190,191,192]. In clinical practice, current evidence supports prescribing at least 150–300 min/week of moderate-intensity aerobic exercise or 75–150 min/week of vigorous-intensity exercise, combined with resistance training performed 2–3 days/week involving the major muscle groups [207]. Exercise intensity should be individualized according to functional capacity, comorbidities, and cardiovascular risk, with gradual progression to optimize adherence and minimize adverse events [130]. Such precision-based frameworks align with contemporary concepts of personalized medicine and may enhance long-term treatment success [187]. A practical summary of exercise prescription, including exercise modality, typical dose, target population, expected clinical outcomes, safety considerations, and level of evidence, is presented in Table 1.

Exercise also exhibits substantial synergistic potential when combined with dietary interventions and emerging pharmacotherapies [207,208,209]. Nutritional strategies that improve energy balance and reduce metabolic stress may amplify exercise-induced adaptations, while pharmacological agents targeting obesity, glucose dysregulation, dyslipidemia, or cardiovascular risk factors may complement the systemic benefits of physical activity [208,209].

**Figure 4:**
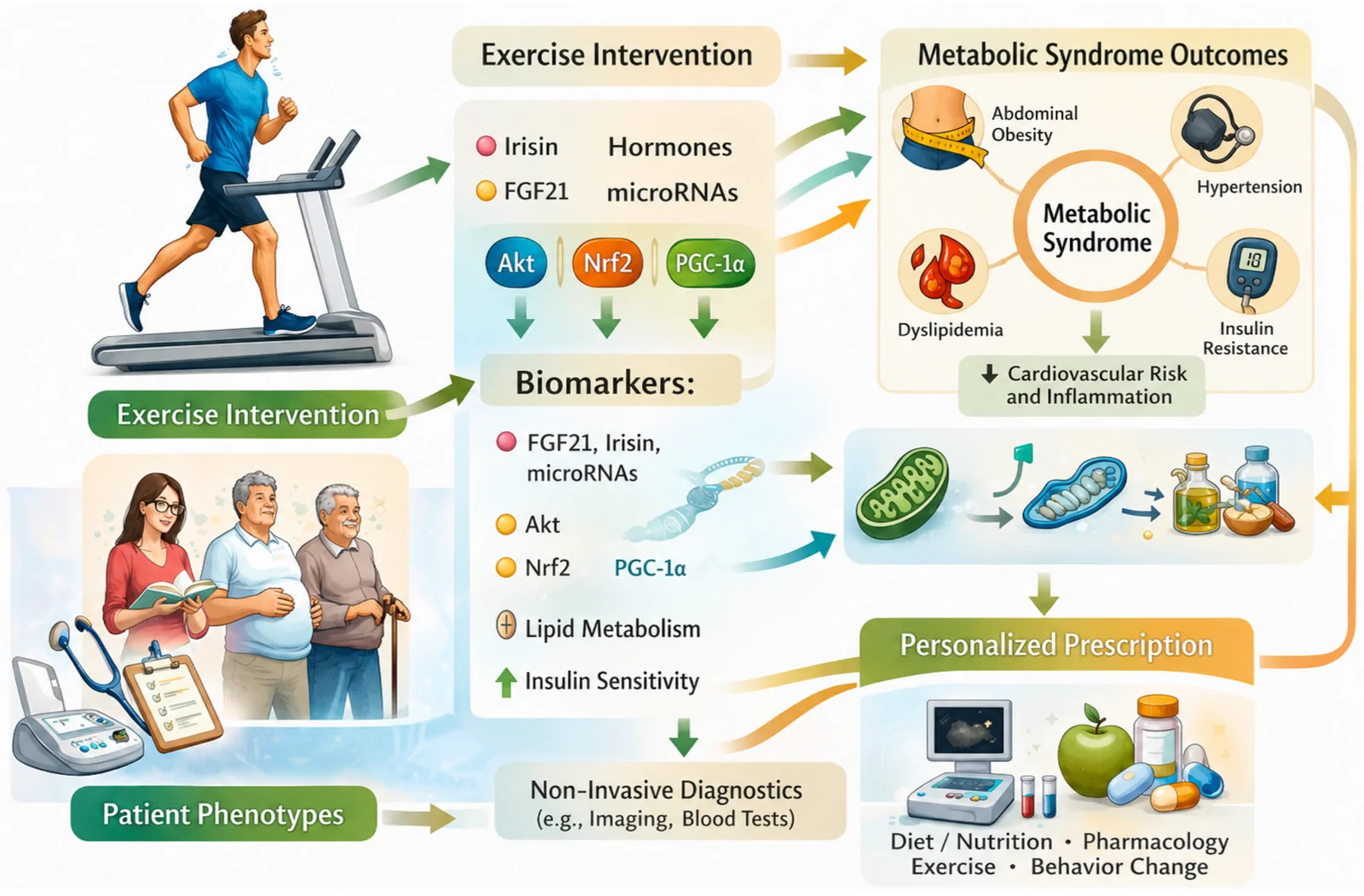
Translational and clinical perspectives from molecular mechanisms to therapeutic applications. Source: own elaboration.

Finally, Figure 4 summarizes the clinical and translational implications of exercise in metabolic syndrome.

## 7. Limitations and Future Directions

Despite considerable advances in understanding the molecular mechanisms through which exercise improves MetS, several methodological and conceptual limitations remain. Significant heterogeneity in study populations, diagnostic criteria, exercise protocols, and outcome measures complicates comparisons across studies and limits the generalizability of findings. Individuals with MetS present diverse clinical phenotypes characterized by varying degrees of obesity, insulin resistance, dyslipidemia, hypertension, and physical deconditioning, which may influence both molecular and physiological responses to exercise. Furthermore, many mechanistic insights originate from animal models or cellular studies that only partially reproduce the complex metabolic, endocrine, immune, and behavioral factors influencing exercise adaptations in humans.

Another important limitation is the lack of standardized exercise prescriptions and molecular assessment protocols. Aerobic, resistance, high-intensity interval training (HIIT), and combined exercise modalities activate overlapping yet distinct signaling pathways, including AMPK, PGC-1α, PI3K/Akt, Nrf2, and CaMK. However, differences in exercise intensity, volume, frequency, and duration often result in heterogeneous outcomes. In addition, acute and chronic exercise induce distinct molecular responses, and many studies lack comprehensive temporal profiling of exercise-induced adaptations. This limitation is particularly relevant for the assessment of circulating biomarkers such as myokines, adipokines, inflammatory mediators, oxidative stress markers, and exercise-responsive microRNAs, whose concentrations are influenced by multiple physiological and environmental factors.

Future progress will require the integration of multi-omics approaches, including transcriptomics, proteomics, metabolomics, epigenomics, and microbiome analyses, within well-controlled human exercise studies. These technologies may provide a more comprehensive understanding of the molecular networks underlying exercise responsiveness and help identify biological signatures associated with favorable cardiometabolic adaptations. Nevertheless, the implementation of multi-omics strategies in clinical and exercise settings remains expensive and methodologically demanding, highlighting the need for standardized analytical pipelines and reproducible research frameworks.

Future research should also prioritize longitudinal and translational studies capable of linking molecular adaptations with clinically meaningful outcomes. The identification of biomarkers predictive of exercise responsiveness could facilitate the development of precision exercise medicine for individuals with MetS. In this context, personalized exercise prescriptions may represent a promising future strategy, allowing exercise modality, intensity, volume, and progression to be tailored according to individual metabolic phenotypes. Molecular characteristics such as insulin signaling efficiency, AMPK activation capacity, mitochondrial adaptability, inflammatory status, oxidative stress burden, and exercise-responsive microRNA profiles may help predict therapeutic responsiveness and optimize cardiometabolic outcomes. Such individualized approaches could maximize improvements in insulin sensitivity, glucose homeostasis, lipid metabolism, body composition, and cardiovascular risk reduction.

Ultimately, advancing exercise-based therapies for MetS will require interdisciplinary collaboration among exercise physiologists, molecular biologists, endocrinologists, cardiologists, nutrition scientists, and public health specialists. The development of standardized, reproducible, and clinically applicable frameworks integrating molecular biomarkers with exercise prescription may facilitate the translation of mechanistic discoveries into personalized interventions capable of addressing the heterogeneity and complexity of metabolic syndrome.

## 8. Conclusions

MetS is a multifactorial cardiometabolic disorder driven by interconnected alterations in insulin signaling, mitochondrial function, inflammatory pathways, endothelial homeostasis, and inter-organ communication. Growing evidence indicates that these mechanisms are not isolated events but components of an integrated pathophysiological network that promotes metabolic dysfunction and cardiovascular risk.

Exercise represents a unique systems-level therapeutic intervention capable of simultaneously modulating many of these disease-driving pathways. Through the activation of molecular regulators such as AMPK, PGC-1α, Akt, and Nrf2, exercise improves metabolic flexibility, enhances mitochondrial function, attenuates chronic inflammation, and restores physiological communication among metabolic organs. These adaptations provide a mechanistic basis for the well-established clinical benefits of aerobic, resistance, and high-intensity interval training in individuals with metabolic syndrome.

Current advances in biomarker discovery, multi-omics technologies, and digital health tools support the emergence of precision exercise medicine as a promising strategy for individualized cardiometabolic care. Future research should focus on identifying molecular predictors of exercise responsiveness and refining personalized exercise prescriptions to maximize therapeutic efficacy and long-term health outcomes in metabolic syndrome.

## Figures and Tables

**Table 1:** Exercise prescription recommendations for MetS.

Exercise Modality	Typical Dose	Outcome	Safety
Aerobic training	150–300 min/week moderate; 75–150 min/week vigorous	Insulin sensitivity; CRF; BP; lipids	Individualization; screening
Resistance training	2–3 days/week; 1–3 sets; 8–12 reps	Strength; muscle mass; glucose control	Technique; load adjustment
HIIT	2–3 days/week; intervals	VO_2_max; insulin sensitivity; body composition	Stable patients; supervision
Combined training	Aerobic (3–5 sessions/week) + resistance (2–3 sessions/week)	Cardiometabolic health; body composition	Individual progression

Source: adaptation of Bull et al. [207]. BP, blood pressure; CRF, cardiorespiratory fitness; HIIT, high-intensity interval training.

## Data Availability

No new data were created or analyzed in this study. Data sharing is not applicable to this article.
